# When the central integrator disintegrates: A review of the role of the thalamus in cognition and dementia

**DOI:** 10.1002/alz.13563

**Published:** 2023-12-02

**Authors:** J. Matthijs Biesbroek, Marieke G. Verhagen, Stefan van der Stigchel, Geert Jan Biessels

**Affiliations:** ^1^ Department of Neurology UMC Utrecht Brain Center University Medical Center Utrecht Utrecht The Netherlands; ^2^ Department of Neurology Diakonessenhuis Hospital Utrecht The Netherlands; ^3^ VIB Center for Brain and Disease Leuven Belgium; ^4^ Department of Neurosciences Katholieke Universiteit (KU) Leuven Leuven Belgium; ^5^ Department of Experimental Psychology Helmholtz Institute Utrecht University Utrecht The Netherlands

**Keywords:** cognition, dementia, functional anatomy, neuroanatomy, thalamus

## Abstract

The thalamus is a complex neural structure with numerous anatomical subdivisions and intricate connectivity patterns. In recent decades, the traditional view of the thalamus as a relay station and “gateway to the cortex” has expanded in recognition of its role as a central integrator of inputs from sensory systems, cortex, basal ganglia, limbic systems, brain stem nuclei, and cerebellum. As such, the thalamus is critical for numerous aspects of human cognition, mood, and behavior, as well as serving sensory processing and motor functions. Thalamus pathology is an important contributor to cognitive and functional decline, and it might be argued that the thalamus has been somewhat overlooked as an important player in dementia. In this review, we provide a comprehensive overview of thalamus anatomy and function, with an emphasis on human cognition and behavior, and discuss emerging insights on the role of thalamus pathology in dementia.

## 1 INTRODUCTION

Dementia is a clinical syndrome, often with devastating consequences for patients and their relatives. By definition, patients with dementia have acquired cognitive impairment interfering with their ability to function independently in activities of daily living.^1^ The clinical phenomenology of dementia is very broad, ranging from impairment in various cognitive functions^1^ (eg, memory, language, attention, executive functioning, visuospatial functions) to neuropsychiatric symptoms^1^, ^2^ (eg, mood and affective disorders, anxiety, agitation, apathy), which may be accompanied by sensory illusions and hallucinations,^2^ motor symptoms such as parkinsonism, gait disorders,^3^ and sleep disorders.^2^, ^3^

Recognizing specific patterns and combinations of cognitive and behavioral symptoms forms the cornerstone of the clinical classification of dementia syndromes. This “pattern recognition approach” in diagnosing dementia is operationalized in established diagnostic clinical criteria for several dementia types.^1^, ^3^, ^4^ However, cognitive and behavioral symptoms reflect the location of brain injury and not necessarily a specific etiology. Therefore, etiological diagnoses in dementia increasingly rely on biomarkers that reflect the actual underlying biology of disease.^5^ Yet, correlating clinical symptoms with the location of brain injury remains critical in case of visible lesions (eg, vascular lesions, focal atrophy, or hypometabolism) to ascertain whether they should be considered causal or merely a coincidental finding.^4^ In addition, in less localized conditions such as Alzheimer's disease (AD), pathological lesions are associated with distinct clinical phenotypes depending on the topography of brain injury, for example, amnestic syndrome, posterior cortical atrophy, corticobasal syndrome, logopenic variant of primary progressive aphasia, and frontal variant (behavioral/dysexecutive) of AD.^6^

Most cognitive functions and behaviors depend on large distributed brain networks, rather than single locations. Accumulating research has provided a detailed picture of cognitive brain anatomy. Herein, we would like to highlight the important role of the thalamus, which is at the center of numerous brain circuits involved in cognitive functioning and regulation of mood and behavior. Accordingly, thalamus pathology can cause a broad range of cognitive and behavioral symptoms, the nature of which depends on its location in relation to specific thalamic nuclei and resulting disconnections. In the context of dementia, thalamus pathology is increasingly recognized as an important contributor to cognitive decline and behavioral symptoms.

In this review, we provide a didactic primer on thalamus anatomy and function, and thalamic pathology in the context of dementia. By first addressing functional thalamus anatomy and then shifting to the context of dementia, we aim to provide the reader with fundamental knowledge on thalamus function, which forms the basis for understanding how thalamus pathology causes cognitive and behavioral symptoms. We therefore first provide a historical perspective on the role of the thalamus in brain function (section 2), a brief introduction to the fundamental concepts of thalamus anatomy and neurophysiology (section 3), and a comprehensive reference work that provides an in‐depth discussion of each thalamic nucleus and its connectivity and function (section 4). We then zoom in on one specific aspect of thalamus function (ie, selective attention and sensory integration) to further illustrate the concept that thalamic nuclei form part of large brain networks in which they not only relay information but also integrate, process, and modulate inputs (section 5). Subsequently, we focus on the role of thalamus pathology in different types of dementia, including AD, Lewy body disease, and vascular cognitive impairment (section 6). In this section, we integrate information from the prior sections on thalamus anatomy and function with evidence for a role of thalamus pathology in cognitive and behavioral decline in the context of dementia. We end with a discussion of clinical implications and future directions (section 6.3) and with a summary (section 7).

**RESEARCH IN CONTEXT**

1. **Systematic review**: We reviewed the literature on thalamus anatomy and function, aiming to provide a didactic primer that provides clinicians and scientists with a sound basis for understanding thalamus function and the consequences of thalamic injury in the context of dementia.
2. **Interpretation**: The thalamus is a central processor and integrator of inputs from across the entire brain, including sensory, cortical, subcortical, and limbic regions. In this review, we discuss and bring together fundamental concepts of thalamus anatomy, circuitry, connectivity, and function (sections 2 through 5) with evidence for an important role of thalamus pathology in dementia (section 6) and discuss clinical implications and possible future directions.
3. **Future directions**: The thalamus is critical for nearly all aspects of human cognition and behavior. The thalamus merits attention in dementia research, given its important contribution to cognitive and behavioral symptoms and as a potential treatment target.

## 2 HISTORICAL PERSPECTIVE

### 2.1 Neuroanatomy of human cognition — cortical versus subcortical

In the 19th century, Broca and Wernicke were among the first to document empirical evidence for the concept that the brain has a functionally specialized architecture.^7^, ^8^ By studying patients with acquired brain lesions, they identified both cortical and subcortical anatomical structures that are critically involved in language. Thus, the role of cortical, as well as subcortical, brain structures in cognition received early recognition. Yet, in the early 20th century, attention shifted to cortical localization of cognitive functions, largely ignoring subcortical connections.^9^, ^10^ Interest in subcortical connections was revived when Geschwind published his paper on “disconnection syndromes” in 1965.^11^ Over time, concepts of cortical specialization and subcortical connections were reintegrated and further refined into the current notion that higher brain functions are the result of an integrated network involving cortical regions, subcortical nuclei, and (sub)cortical connections.^9^, ^10^

### 2.2 The thalamus

The first mention of the thalamus has been traced back to Galen in the second century AD.^12^ Galen dissected the optic nerve and optic tract to its termination in the lateral geniculate nucleus (LGN) of the thalamus. It is believed that he used the Greek word *thálamos*, which means “central chamber,” to refer to the descending part of the lateral ventricle that is adjacent to the LGN instead of to the thalamic nucleus itself. In the 17th century, Thomas Willis created anatomical drawings in which he treated the thalamus as a central brain region that connected the brain stem and cerebral hemispheres.^12^ Thus, Willis continued to use the name “thalamus,” albeit to refer to a different structure than Galen probably had in mind. In the late 19th century, Luys created a functional model of the thalamus when he published his model of sensorimotor processes of cerebral activity, in which he described the function of the thalamus as a relay center of sensory input to other brain regions.^12^ This notion of the thalamus as a relay center has long been the dominant view, and the thalamus has accordingly been referred to as “the gateway to the cortex.”^13^ We now know that the thalamus does not only relay sensory input (as the term “gateway to the cortex” suggests) but also receives input from the cortex and limbic systems. In the current network models of brain function, the thalamus therefore plays a central role as it receives, integrates, and modulates input from all five sensory systems, the cortex, basal ganglia, limbic system, brain stem nuclei, and cerebellum. By modulating and integrating these inputs, the thalamus is critically involved in many brain functions, ranging from arousal and attention, which is at the core of performing any given task, to more specific aspects of cognitive functioning, mood, and behavior.

## 3 FUNDAMENTAL CONCEPTS

### 3.1 Thalamic subdivisions

The thalamus can be divided into a number of nuclei consisting of cell populations that receive specific afferent input and project to a distinct (group) of brain regions. This division into nuclei is mainly based on *post mortem* and experimental studies from the late 19th and first half of the 20th century in which axons were traced to large thalamic cell groups.^12^ This classical approach has formed the basis of our understanding of the functional neuroanatomy of the thalamus. For example, retinal projections can be traced through the optic tract to neurons in the LGN of the thalamus, and these neurons in turn have projections that were traced to the occipital cortex, leading to the conclusion that the LGN is a relay center for visual input to the primary visual cortex. Thus, the categorization of the major thalamic nuclei is based on the identification of thalamic cell populations that share the same input and output and are therefore assumed to form a specific functional module.

This classical view of the thalamus still has its merits, even though studies from recent decades have revealed a detailed picture of thalamus anatomy that is far more complex.^12^ We now know that cell populations within thalamic nuclei are diverse and that these neurons have complex connectivity patterns, not only to other parts of the brain, but with other thalamic nuclei as well. These connectivity patterns are difficult to untangle completely, even with modern research techniques. In this light, the thalamic subdivisions (presented in section 4, “Functional anatomy”) represent a simplification that does not entirely do justice to the complexity of the thalamus. However, it remains the most useful system for a practical classification of thalamus anatomy and function, and it will therefore form the basis for this review of functional thalamus anatomy.

### 3.2 Thalamic circuitry – relays and modulation

Thalamic nuclei can be further classified according to their circuitry (ie, input, output, and internal functional architecture). The first type of thalamic circuitry is a relay of sensory input to the cortex, which is referred to as a “first‐order relay.”^13^ A thalamic nucleus with this type of circuitry is usually called a “principal” or “relay nucleus.”^13^, ^14^ The second type of circuitry involves input from the cortex and output to a range of possible brain regions including cortex and basal ganglia. This type of circuitry is referred to as a “second‐order relay,” and a thalamic nucleus with such circuitry is called an “association nucleus.”^13^, ^14^ There is debate as to whether thalamic nuclei that receive input from the limbic system should have their own classification (ie, called limbic nuclei) or whether the aforementioned classification of association nuclei should include nuclei with limbic as well as cortical input.^14^ The neurotransmission involved in thalamic nuclei is diverse and includes the neurotransmitters glutamate, GABA, norepinephrine, acetylcholine, serotonin, histamine, dopamine, adenosine, orexin, and a broad range of neuropeptides.^15^, ^16^, ^17^

**FIGURE 1:**
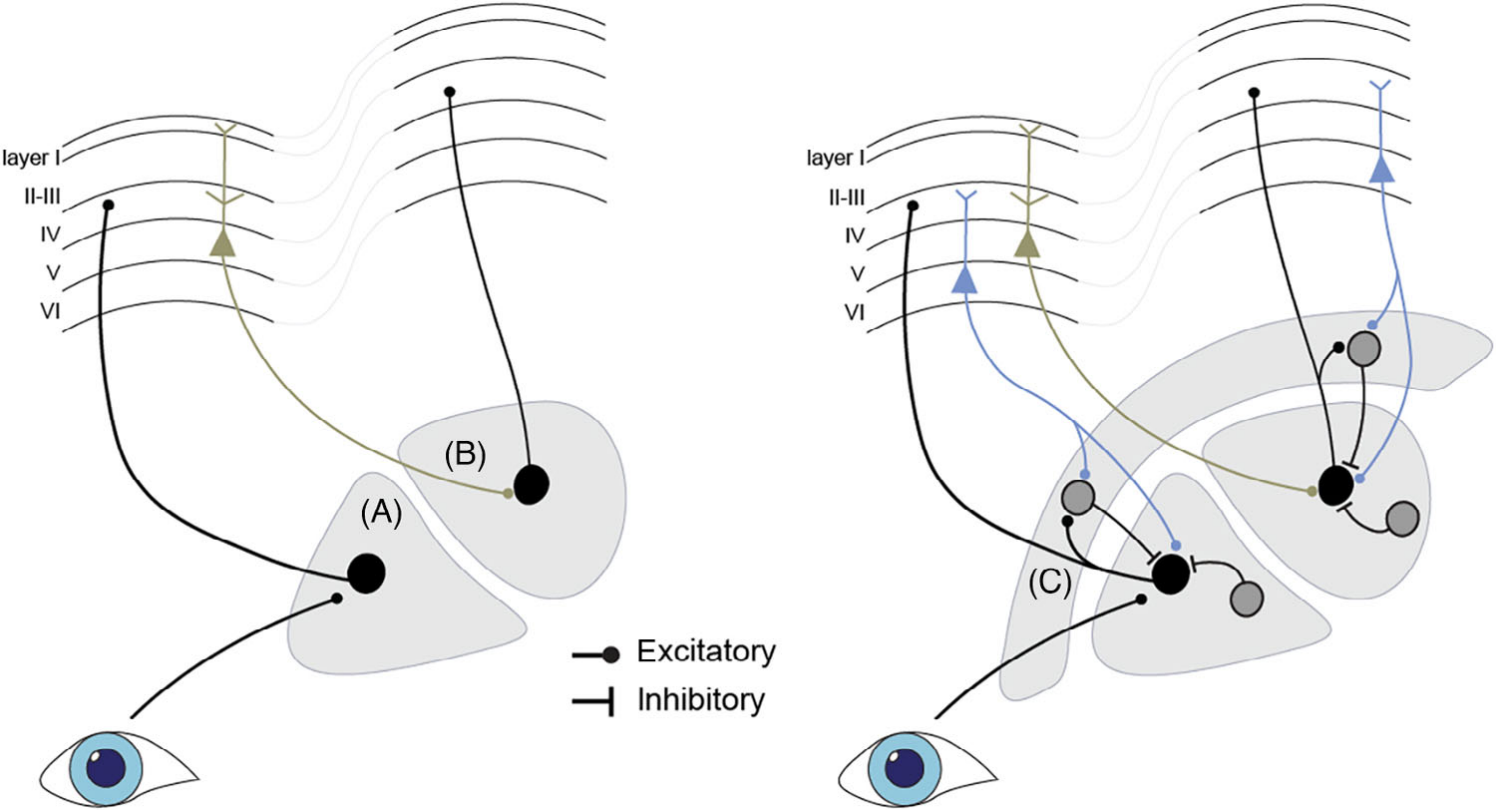
Schematic representation of first‐order and higher‐order relays of thalamic circuitry. The left panel provides a simplified model in which the first‐order relay (A) receives sensory input and projects to the cortex, whereas the higher‐order relay (B) receives cortical input from layer 5 excitatory neurons and projects to another cortical region. The right panel shows a more detailed model incorporating corticothalamic modulation from layer 6 excitatory neurons and inhibitory input from the reticular nucleus (C) and local inhibitory neurons, facilitating complex modulation of signals in both first‐ and higher‐order relays.

Classical models mainly consider thalamic nuclei as relay centers of either sensory or cortical input. In reality, the thalamus contains complex internal circuitry enabling modulation and integration of inputs that is essential for cognitive functioning and behavior.^13^ Modulation of input is known to occur not only in association nuclei but in relay nuclei as well. See, for example, section 5.1.2, in which the LGN (a visual relay nucleus) and its role in modulating visual inputs to facilitate selective attention to visual stimuli is discussed. A schematic illustration of circuitry involved in first‐ and second‐order relays and an example of internal circuitry enabling modulation of inputs is provided in Figure 1.

## 4 FUNCTIONAL ANATOMY

**FIGURE 2:**
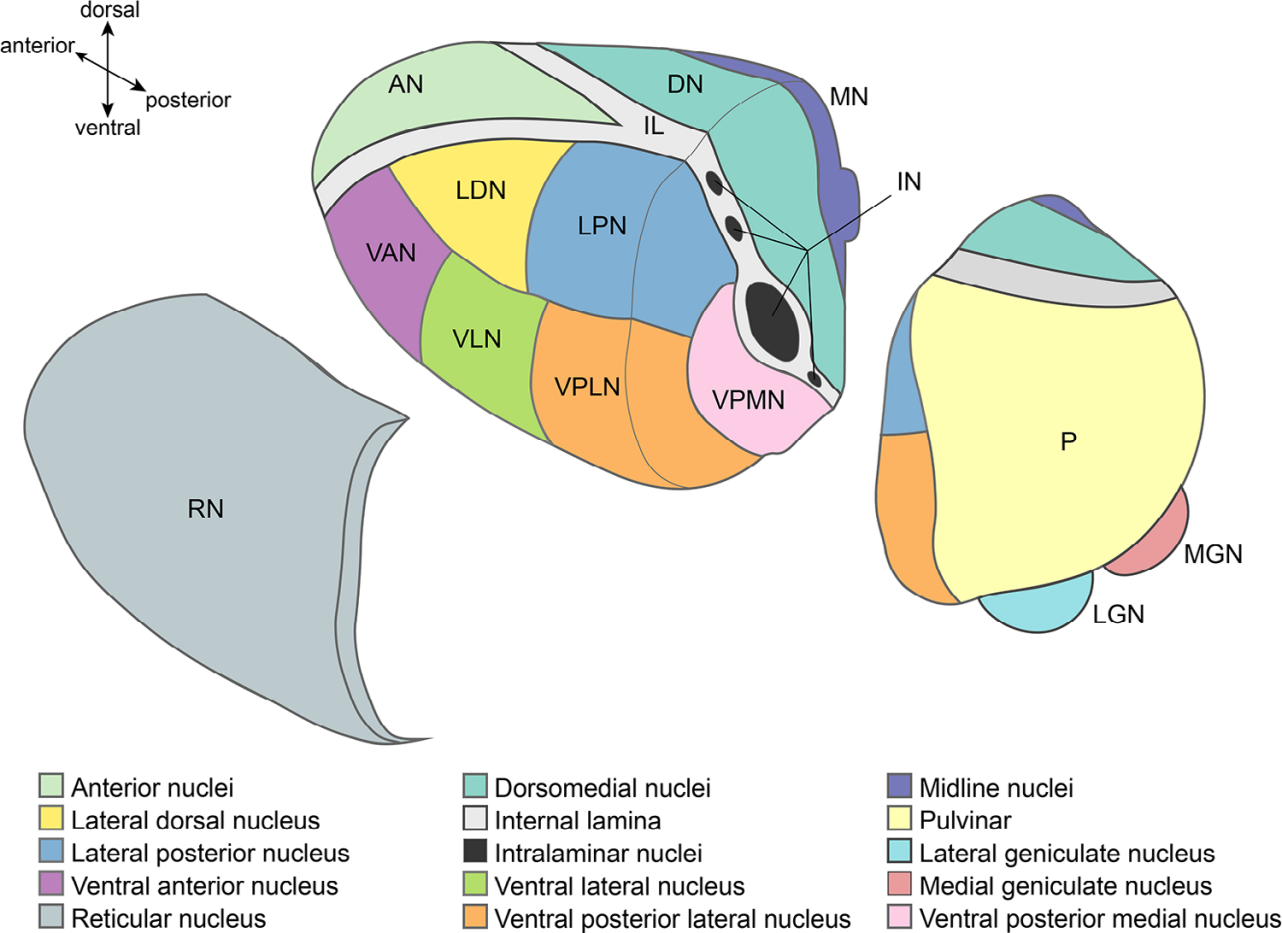
Schematic drawing of thalamus anatomy. The reticular nucleus is separated to reveal the dorsolateral nuclei located underneath, and the posterior part of the thalamus is separated from the anterior part of the thalamus to show the intralaminar nuclei and ventral posterior medial nucleus.

Together with the epithalamus, hypothalamus, and subthalamus, the thalamus forms the diencephalon.^18^ Here we will not further discuss the epithalamus (including the pineal gland and habenula), the subthalamus (including subthalamic nucleus, zona incerta, and nucleus of the field of Forel), and hypothalamus. The third ventricle forms the medial border of each thalamus and the internal capsule its lateral border.^12^, ^13^, ^19^ The thalamus proper consists mostly of gray matter, with interspersed white matter. An overview of thalamus subdivisions is provided in Figure 2. Taking the ovoid structure of the thalamus as a starting point, the Y‐shaped internal lamina, mainly consisting of white matter fibers, divides the thalamus into anterior, dorsomedial, and dorsolateral groups of nuclei. The dorsolateral group can be further divided into nuclei, each of which processes specific sensory or motor input, and several nuclei that integrate sensory input, most notably the pulvinar. Wrapped around the lateral border of the thalamus is the reticular nucleus, which interacts with many of the thalamic nuclei. The internal lamina also contains several small nuclei, the so‐called intralaminar nuclei. Finally, the paramedian part of the thalamus contains a specific group of nuclei referred to as the midline nuclei. Each of these nuclei will be discussed (summary provided in Table 1), focusing on their connectivity and function.

**TABLE 1 alz13563-tbl-0001:** Summary of thalamic nuclei and their function.

Thalamic group	Specific nucleus	Main input	Main output	Function
Anterior nuclei		‐ Fornix, mammillary bodies ‐ Cingulate cortex ‐ Lateral dorsal tegmental nucleus	‐ Fornix, mamillary bodies ‐ Cingulate cortex ‐ Prefrontal cortex	‐ Attention and executive ‐ Learning/memory ‐ Language (fluency and naming) ‐ Mood and behavior
Dorsomedial nuclei	All divisions	‐ Prefrontal cortex ‐ Pallidum ‐ Substantia nigra	‐ Prefrontal cortex	‐ Executive functioning ‐ Memory ‐ Verbal fluency and naming ‐ Mood and behavior
	Parvocellular division	‐ Amygdala ‐ Hippocampal formation ‐ Olfactory bulb and piriform cortex		
Dorsolateral nuclei	Lateral geniculate nucleus	‐ Optic tract	‐ Primary visual cortex	‐ Vision
	Medial geniculate nucleus	‐ Inferior colliculus	‐ Primary auditory cortex	‐ Hearing
	Lateral and inferior pulvinar	‐ Lateral geniculate nucleus ‐ Visual cortex ‐ Superior colliculi	‐ Occipital association cortex ‐ Temporal association cortex	‐ Spatial attention ‐ Visuospatial perception ‐ Visually guided action
	Medial pulvinar	‐ Reciprocal connections with widespread association cortices, cingulate cortex, insula, hippocampus; input from superior colliculi		‐ Multisensory integration and selective attention ‐ A range of complex cognitive functions
	Anterior pulvinar	‐ Reciprocal connections with posterior parietal and secondary somatosensory cortex		‐ Cognitive aspects somatosensation and pain
	Lateral dorsal nucleus	Reciprocal connections with frontal and cingulate cortex and hippocampal formation. Additional input: pretectum, superior colliculus, and lateral geniculate nucleus		‐ Visual/spatial memory
	Lateral posterior nucleus	‐ Visual cortex and colliculi	‐ Parietal cortex	‐ Visual and somatosensory integration ‐ Spatial cognition
	Ventral posterior lateral nucleus	‐ Spinothalamic and medial lemniscus	‐ Postcentral gyrus	‐ Somatosensation trunk and limbs
	Ventral posterior medial nucleus	‐ Trigeminothalamic ‐ Central tegmental tract	‐ Postcentral gyrus ‐ Anterior insular and frontal opercular cortex	‐ Somatosensation face ‐ Gustation
	Ventral anterior nucleus	‐ Nigrostriatal	‐ Premotor and supplementary motor cortex	Motor initiation and planning
	Ventral lateral nucleus Ventral intermediate nucleus	‐ Corticostriatal ‐ Corticocerebellar	Precentral gyrus	Motor planning: integrating cerebellar, cortical, and striatal input
Intralaminar nuclei		‐ Reticular formation ‐ Cerebral cortex ‐ Basal ganglia ‐ Trigeminal and spinothalamic ‐ (Cerebellum)	‐ Cerebral cortex ‐ Basal ganglia ‐ Limbic system	‐ Consciousness and arousal ‐ Sensory‐motor integration
Reticular nucleus		‐ Thalamic nuclei ‐ Basal forebrain ‐ Striatum ‐ Brain stem	‐ Thalamic nuclei ‐ Mesencephalon	‐ Regulating consciousness and arousal ‐ Gating/modulation thalamic output
Midline nuclei		Bidirectional projections with cortical and subcortical limbic structures		‐ Learning and memory ‐ Emotional regulation and affective behavior ‐ Arousal and sleep–wake cycle

### 4.1 Anterior nuclei – mood, emotion, behavior, cognition

The anterior thalamic group can be further divided into the anterior dorsal, anterior ventral, and anterior medial nuclei.^20^ These nuclei have reciprocal connections (ie, both afferent and efferent connections) with the hippocampal formation (via the fornix, mamillary bodies, and mammillothalamic tract) and the cingulate gyrus, and additionally receive input from the tegmentum (lateral dorsal tegmental nucleus) and project to the prefrontal cortex.^18^, ^20^, ^21^ Thus, these nuclei are a critical link in the limbic system (regulating mood, emotion, and behavior) and circuits for learning and memory. Furthermore, these nuclei are involved in attention and executive functioning^20^, ^22^ and several aspects of language, most noticeably verbal fluency and naming.^23^, [Table alz13563-tbl-0001]

### 4.2 Dorsomedial nucleus – mood, emotion, behavior, cognition

The dorsomedial nucleus forms a large part of the thalamus and can be further divided into the magnocellular division, parvocellular division, and lateral (sometimes called paralaminar) division. The dorsomedial nucleus has reciprocal connections with the prefrontal cortex and cingulate cortex. The magnocellular division projects mainly to the medial and orbital frontal cortex, the parvocellular division to the dorsolateral prefrontal cortex, and the lateral division to the frontal eye fields as well as lateral frontal cortex.^19^, ^24^, ^25^, ^26^ Apart from receiving afferents from these frontal cortical regions, all divisions receive input from the pallidum, ventral tegmental area, substantia nigra, and other brain stem regions.^26^ The parvocellular division additionally receives input from the amygdala, parahippocampal formation, and olfactory piriform cortex.^19^, ^25^, ^26^ Of note, olfaction is the only sensory modality for which the thalamus does not receive input from primary sensory neurons, but only from cortical regions.^27^ The dorsomedial nucleus is prominently involved in executive functioning,^28^ including planning, decision‐making, cognitive control, and multitasking,^25^ as would be expected given its strong reciprocal connections with the prefrontal cortex. Furthermore, the dorsomedial nucleus is involved in regulating mood,^29^ motivation, and learning and memory.^25^

### 4.3 Lateral nuclei – sensory processing, motor function, cognition

#### 4.3.1 Lateral and medial geniculate nuclei – visual and auditory relay

The geniculate nuclei are mainly a relay center for visual and auditory input. The LGN receives input from retinal projections (through the optic nerve and optic tract) and relays visual input to the primary visual cortex (calcarine cortex) and surrounding cortices.^18^, ^19^, ^30^ The medial geniculate nucleus receives its main input from the inferior colliculus, which in turn receives input from the cochlear nuclei in the brain stem. The medial geniculate nucleus has a laminated structure, with each lamina receiving auditory input with specific frequencies (ie, higher frequencies are localized medially, lower frequencies laterally) that projects to the primary auditory cortex (ie, Heschl's gyrus).^18^, ^19^, ^31^ A more detailed description of medial geniculate nucleus connectivity is provided elsewhere.^31^ The suprageniculate and limitans nuclei are small thalamic nuclei located in close proximity to the medial geniculate nucleus and are thought to be mainly involved in auditory processing.^32^

#### 4.3.2 Pulvinar – sensory integration, cognition

The pulvinar is the largest thalamic nucleus, taking up approximately 30% of thalamic volume in humans, and is divided in the medial, lateral, inferior, and anterior pulvinar. The pulvinar receives input from multiple sensory modalities, has reciprocal connections with numerous cortical and limbic regions, and is critically involved in sensory integration and many aspects of cognitive functioning, including selective attention, visuospatial processing, and language (more specifically, lexical‐semantic processes^33^).

The lateral pulvinar (in particular the ventrolateral part) and the inferior pulvinar are involved in visual processing. They receive input from the LGN, visual cortex, and superior colliculi and project to occipital and temporal association cortex.^34^ The lateral and inferior pulvinar form a central part of both the ventral stream (for recognizing objects and faces) and the dorsal stream (for visually guided action).^34^

The medial pulvinar in humans is thought to be a recently evolved part of the pulvinar^35^ that has widespread reciprocal connections to the prefrontal cortex, temporal and parietal association cortices, posterior cingulate cortex, insula, and hippocampus.^34^, ^35^ The medial pulvinar is an association nucleus involved in integrating heteromodal input from association cortices and limbic regions, facilitating multisensory integration, attention, and a range of complex cognitive functions.^34^, ^36^, ^37^ The dorsomedial part of the lateral pulvinar has connections to inferior parietal and prefrontal cortices and is therefore functionally more related to the medial pulvinar than to the ventrolateral part of the lateral pulvinar.^38^

The anterior pulvinar mainly has reciprocal connections with the posterior parietal cortex and secondary somatosensory cortex and is involved in cognitive aspects of somatosensation and pain.^36^, ^39^

#### 4.3.3 Ventral posterior nucleus – somatosensation

The ventral posterior nucleus, sometimes also referred to as the ventrobasal complex, is the main relay for somatosensory input and can be further divided into lateral and medial nuclei. The ventral posterior lateral nucleus receives input from the spinothalamic and medial lemniscus pathways.^19^ The ventral posterior medial nucleus receives sensory input from the face (trigeminothalamic tract) and gustatory input from cranial nerves VII, IX, and X (central tegmental tract).^19^ The ventral posterior nucleus projects to the primary somatosensory cortex for sensations of touch, pain, vibration, temperature, and proprioception^18^ and to the gustatory cortex (located mainly in the anterior insular and frontal opercular cortex^40^) for gustation.

#### 4.3.4 Ventral anterior and ventral lateral nucleus – motor function

The ventral anterior nucleus and the ventral lateral nucleus are mainly involved in motor processing. The ventral anterior nucleus receives input from the direct and indirect nigrostriatal pathways and mainly projects to the premotor and supplementary motor cortex.^18^, ^19^, ^41^ The ventral lateral nucleus receives input from corticostriatal and corticocerebellar pathways and projects mainly to the primary motor cortex.^18^, ^19^, ^42^ The ventral lateral nucleus has a somatotopic organization in which the lateral part projects to the leg region of the precentral cortex and the medial part to the head region.^18^ The subregion within the caudal part of the ventral lateral nucleus, known as the ventral intermediate nucleus, relays cerebellar input and is an important target for deep brain stimulation for treating tremors.^43^ In a simplified model incorporating the functions of these motor nuclei, (1) the ventral anterior nucleus is involved in the initiation and early planning of motor programs by providing nigrostriatal input to the premotor and supplementary motor cortices, and (2) the ventral lateral nucleus is involved in fine‐tuning the motor programs generated by premotor and supplementary motor cortex by integrating input from corticostriatal and corticocerebellar pathways and transferring the motor program to the primary motor cortex for execution.

#### 4.3.5 Dorsal nuclei – (spatial) memory, sensory integration

The lateral dorsal and the lateral posterior nuclei are located dorsally in the lateral thalamus and are primarily involved in cognitive functioning. The most anterior of these two nuclei is the lateral dorsal nucleus, which works in concert with the anterior thalamic nuclei^44^ (section 4.1). The lateral dorsal nucleus has reciprocal connections with the frontal and cingulate cortex (including retrosplenial cortex) and hippocampal formation, similar to the anterior thalamic nuclei.^44^ A major difference in terms of input between the anterior thalamic nucleus and the lateral dorsal nucleus is that the first receives afferents from the mammillary bodies,^18^, ^44^ whereas the latter receives mainly afferents from visual structures including the pretectum, superior colliculus, and LGN.^44^, ^45^ Accordingly, the lateral dorsal nucleus is thought to be critical for learning from visual input and spatial memory in particular.^44^

The nomenclature of the lateral posterior nucleus is inconsistent, given that the terms oral pulvinar nucleus and anterodorsal pulvinar nucleus are sometimes used to refer to the same structure.^34^, ^46^ Furthermore, the nomenclature in mammals is different for primates compared to non‐primates. In non‐primates, who have a less developed pulvinar than primates, it is common to refer to a large part of or the entire pulvinar complex as the lateral posterior nucleus.^47^ In primates, the lateral posterior nucleus is considered a separate nucleus that is closely related to the pulvinar in terms of connectivity and function.^19^ It receives input from the visual cortex and colliculi,^13^ projects to the parietal cortex, and is involved in visual and somatosensory integration and spatial cognition.^19^

### 4.4 Intralaminar nuclei and reticular nucleus – arousal and attention

The intralaminar nuclei form part of the ascending reticular activating system (ARAS) and are thus critical for consciousness and arousal. The ARAS mainly originates from the reticular formation in the pons (other nuclei contributing to the ARAS include the locus coeruleus, dorsal raphe, median raphe, pedunculopontine nucleus, parabrachial nucleus), ascends through the mesencephalic tegmentum just posterior to the red nucleus, and terminates on the intralaminar nuclei.^48^ From the intralaminar nuclei, the input from the brain stem is further processed and relayed to the cortex. Thus, the intralaminar nuclei connect the ARAS with the cerebral cortex. Bithalamic lesions (eg, bithalamic infarcts) involving the intralaminar nuclei can result in coma, and less extensive lesions in either the left or right thalamus may cause less severe deficits in consciousness, arousal, and attention.

The intralaminar nuclei can be further divided into a posterior cluster, including the centromedian and parafascicular nuclei, and an anterior cluster including the paracentral, central lateral, and central medial nuclei.^14^, ^19^ Apart from being part of the ARAS, the intralaminar nuclei receive input from a large number of regions, including cerebral cortex, basal ganglia, substantia nigra, cerebellum, trigeminal nuclei, and the spinothalamic tract. All intralaminar nuclei have efferent projections to the striatum and to varying locations of the cerebral cortex. The centromedian nucleus additionally has efferent projections to the amygdala and cortical limbic sites.^14^ Apart from their role in regulating consciousness, arousal, and attention, the intralaminar nuclei are involved in sensory‐motor integration and pain regulation. In particular the centromedian nucleus has been recognized as a potential target for deep brain stimulation for the treatment of movement disorders,^49^ chronic pain,^50^ and epilepsy.^51^

The reticular nucleus is a relatively thin nucleus wrapped around nearly the entire anterior and lateral surface of the thalamus. A thin layer of white matter called the external lamina separates the reticular nucleus from the other thalamic nuclei. The reticular nucleus is unique in the sense that it is believed to have no efferent projections to the cortex and only provides GABAergic output to other thalamic nuclei and to the mesencephalic tegmentum.^19^, ^52^ Its main input comes from other thalamic nuclei, striatum, basal forebrain, and brain stem nuclei including the ARAS. The reticular nucleus has a regulatory and “gating” function for activity in nearly all the other thalamic nuclei and, together with the intralaminar nuclei, modulates ARAS activation of thalamocortical networks.^52^ Thus, the reticular nucleus is critical for maintaining and regulating consciousness and attention.

### 4.5 Midline nuclei – memory, mood, behavior

The left and right thalamus are divided by the third ventricle and connected by a small commissure (the interthalamic adhesion) that bridges the third ventricle. The midline nuclei are located underneath the ependyma of the third ventricle within the most median part of the thalamus and within the interthalamic adhesion (Figure 2). These nuclei include the rhomboid, reuniens, paraventricular, and paratenial nuclei.^14^, ^19^ The midline nuclei are part of the limbic system. The reuniens and rhomboid nuclei mainly have bidirectional projections with the hippocampus and limbic cortical structures, particularly the medial prefrontal cortex including anterior cingulate cortex, and are thus critically involved in learning and memory, as well as affective aspects of memory. The paraventricular and paratenial mainly project to limbic subcortical structures, such as the amygdala and nucleus accumbens, and are involved in arousal, emotional regulation, affective behavior, goal‐directed behavior, and sleep–wake cycles.^14^, ^53^

## 5 (SELECTIVE) ATTENTION AND SENSORY INTEGRATION

In the preceding sections, the various subregions of the thalamus were linked to numerous cognitive functions. Here, we zoom in on a specific cognitive function to illustrate how thalamic nuclei form part of large brain networks and show that these nuclei do not only relay information but also process, integrate, and modulate inputs within this network. We provide an in‐depth discussion of the role of the thalamus in selective attention and sensory integration, which play a vital role in many aspects of human cognition and behavior.

### 5.1 Selective attention

Selective attention refers to the cognitive process of directing processing resources to behaviorally relevant stimuli while suppressing irrelevant information.^54^, ^55^, ^56^, ^57^ Stimulus detection and discrimination are improved with selective attention,^55^ enabling us to selectively attend to relevant physical objects and their individual parts in our environment, but also to items from our memory, while ignoring other information.

Traditionally, it was thought that neural mechanisms for selective attention reside solely within the cortex.^55^ However, emerging evidence has indicated that subcortical structures, including the thalamus, play a significant role.^55^, ^56^ In recent decades, more attention has been drawn to interactions between cortical and subcortical brain structures in supporting complex forms of voluntary activity in humans, including selective attention.^58^ Network models of arousal and attention now include a central role of thalamic nuclei in tonic and phasic arousal processes.^59^ In tonic arousal, thalamic nuclei, particularly those in the intralaminar and midline regions, contribute to the maintenance of baseline wakefulness. They relay sensory information to the cerebral cortex, helping to sustain the state of readiness for processing sensory input. This way, thalamic nuclei help in preventing sensory overload. In phasic arousal, thalamic nuclei are involved in rapid alerting responses. When a salient or novel sensory stimulus is detected, they rapidly relay sensory information to the cortex, initiating a phasic arousal response.^60^ Furthermore, these nuclei contribute to selective attention by directing sensory information to the appropriate cortical areas, helping to orient attention. Altogether, thalamic nuclei play a pivotal role in balancing the brain's overall state of alertness and responsiveness to sensory information, acting as a “gatekeeper” that regulates the sensory input of the cortex.

The following subsections present a more detailed discussion of the role of specific thalamic nuclei in (selective) attention, together with an in‐depth discussion of neurophysiological mechanisms that are involved in thalamic regulation of arousal and attention.

#### 5.1.1 Reticular nucleus

Interposed between the thalamus and the cortex, the reticular nucleus intercepts and regulates communication between these two structures (section 4.4).^54^, ^57^ Because the reticular nucleus receives unidirectional projections from the cortex and has bidirectional connections with the thalamus, it is in a central position to modulate the flow of information between the two areas. Through this open‐loop connection, the circuit is postulated to facilitate lateral inhibition, which may allow reticular nucleus cells to modulate thalamocortical cells in such a way as to amplify relevant stimuli to cortical areas while suppressing irrelevant stimuli.^61^, ^62^ The reticular nucleus therefore represents the gate through which sensory information from other thalamic nuclei is relayed to the cortex. This gatekeeping role of the circuitry of the reticular nucleus depends on its physiological state. Whether the reticular nucleus is active depends largely on cholinergic and monoaminergic inputs from the brain stem that regulate the membrane potential of neurons of the reticular nucleus.^57^ By fine tuning the excitability of its neurons, the reticular nucleus allows for the functional flexibility it needs to selectively attend to relevant stimuli in a constantly changing environment. This explains its critical role in maintaining and regulation consciousness and attention.

#### 5.1.2 Lateral geniculate nucleus

The LGN is mostly involved with visual input and passes this input on to the primary visual cortex and surrounding cortices (section 4.3.1). In various attention tasks in functional magnetic resonance imaging (fMRI) studies,^63^ it has been revealed that selective attention affects visual processing not only in cortical levels but also in the LGN in at least three different ways.^64^ Neural responses in the LGN were greater to stimuli that were attended versus to stimuli that were ignored, commonly termed attentional response enhancement. Additionally, the magnitude of the response to ignored stimuli depended on the load of attentional resources engaged elsewhere. Finally, in the absence of a visual stimulus and in anticipation of a new stimulus, neural baseline activity in the LGN increased. In agreement with these human fMRI findings, neurophysiological studies in animals complement the evidence for the role of LGN in attentional modulation.^65^ Macaques were trained to covertly attend to one of two spatial locations and had to make a perceptual judgment on luminance change, demanding selective attention to one spatial location. In this cued‐attention task, LGN neurons showed an increased visual response when its receptive field coincided with the attended location compared to the unattended location.^66^ Therefore, evidence from human fMRI as well as monkey electrophysiological studies are consistent with the view of the LGN's involvement in selective attention.

#### 5.1.3 Pulvinar

Several lines of evidence have shown that the pulvinar should be considered a subcortical component of the attention network.^64^, ^67^ The pulvinar is connected to other cortices in the attention network, such as the prefrontal cortex, posterior parietal cortex, and superior colliculus. Neurons in the pulvinar respond selectively to visual stimulus features such as color, orientation, and motion,^68^ suggesting that the structure receives input from multiple cortical areas. Importantly, it has been shown that selective attention increases BOLD responses^69^, ^70^ and glucose uptake^71^ in the pulvinar.

Lesion studies have shown deficits in engaging attention to the contralesional visual field^72^, ^73^, ^74^, ^75^ and poor filtering of distractors.^76^, ^77^ Furthermore, on the basis of the connectivity of the human pulvinar, it might further serve as a nexus for integrating cortical control of voluntary (top‐down) eye movements with reflexive (bottom‐up) eye movements generated by the superior colliculus. Indeed, various studies have found small, but consistent, deficits in the control of eye movement in patients with a lesion to the pulvinar.^72^, ^78^, ^79^

### 5.2 Sensory integration

In daily life our brain constantly receives information about the world via multiple sensory modalities. Different types of sensory information are processed along various sensory‐specific pathways and subsequently unified into one percept we can make sense of. Previously, it was thought that multisensory integration happens only at the level of the cortex. However, the last years it has become apparent that thalamic structures are also involved in integrating sensory information.^37^

Although the pulvinar is mostly associated with visual processes,^68^, ^80^ electrophysiological studies have shown neural activation in response to more than one sensory modality.^81^ In one study from 2009, retrograde neuronal tracers were injected into the auditory, somatosensory, and premotor cortex of macaques, and it was found that the pulvinar exhibited the most overlap of different retrogradely labeled neurons.^82^ Based on this, the authors concluded that the pulvinar played an important role in multisensory integration. In human fMRI studies, an improved classification of multimodal (ie, audiovisual) compared to unimodal (ie, either auditory or visual) emotional stimuli was linked to enhanced fMRI signals in multisensory zones of cortical as well as subcortical areas, including the thalamus.^83^ In another fMRI study it was found that a task‐irrelevant auditory stimulus increased perceptual sensitivity in multisensory cortical areas, but most importantly also in sensory‐specific lateral geniculate body (LGB) and the auditory thalamus.^37^ fMRI studies such as these provide clear evidence for the involvement of sensory‐specific thalamic structures in multisensory integration.

## 6 THE ROLE OF THALAMUS PATHOLOGY IN DEMENTIA

Given its function as a central integrator of sensory, cortical, and limbic inputs, thalamus pathology can be highly relevant in explaining cognitive and behavioral symptoms in patients with dementia. The next section provides a brief summary of evidence for the role of thalamus pathology in dementia and possible clinical implications and future directions.

### 6.1 Thalamus pathology in specific dementia etiologies

#### 6.1.1 Alzheimer pathology

According to the Braak criteria for staging Alzheimer pathology, stages I and II indicate presence of neurofibrillary tangles in entorhinal cortex, stages III and IV involvement of limbic regions such as the hippocampus, and stages V and VI extensive neocortical involvement.^84^ Although thalamic structures are not explicitly incorporated in the Braak staging criteria, neuropathological studies do report neurofibrillary tangle deposition in several specific thalamic nuclei, particularly the limbic nuclei (i.e, anterior thalamic nuclei, midline nuclei, and centromedian nucleus) as early as Braak stages III and IV.^84^ In particular, the anterior thalamic nuclei are affected. These limbic thalamic nuclei, in particular the anterior thalamic nuclei, have strong reciprocal connections with the hippocampus (through fornix and mammillary bodies) and retrosplenial cortex and play a critical role in learning and memory (section 4.1). Several positron emission tomography and structural MRI studies have demonstrated that hypometabolism and atrophy of both the thalamus and connected retrosplenial cortex occurs in early stages of AD.^85^, ^86^ Along the same line, a recent study performing fMRI and structural imaging in patients with amnestic mild cognitive impairment and healthy controls found structural changes in thalamocortical connections to posterior default mode network (DMN) nodes (ie, posterior cingulate cortex and inferior parietal lobe) in patients with MCI, which correlated with reduced memory performance.^87^ The DMN is a large distributed network involved in cognitive functioning, and DMN perturbations have been demonstrated in early stages of AD and have been associated with cognitive decline.^88^ The anterior and dorsomedial thalamic nuclei are of central importance for the functioning of this network.^89^ Another recent study in cognitively asymptomatic individuals demonstrated an association between higher amyloid levels in the thalamus and thalamic structural abnormalities and between subcortical amyloid levels and lower performance on episodic memory tasks.^90^ Taken together, limbic thalamic nuclei (1) are critically involved in many aspects of cognition and behavior, including episodic memory and learning (section 4 of this review); (2) are affected during early stages of AD, as early as the hippocampus; and (3) AD pathology in these nuclei has been linked with structural and metabolic changes in both the thalamus and widespread brain networks critical for cognition (including the DMN) in patients with (pre)clinical AD. These lines of evidence implicate the thalamus as a relevant structure in AD and suggest that thalamic injury is an important contributor to cognitive and behavioral impairment in AD.

#### 6.1.2 Alfa‐synucleinopathies

Alfa‐synuclein deposition in the form of Lewy bodies and Lewy neurites are the neuropathological hallmark of Parkinson's disease (PD) and Lewy body dementia (LBD). According to the Braak neuropathological staging system for PD, thalamic pathology may start to accumulate during stage IV.^91^ There is evidence that alfa‐synuclein pathology occurs in specific thalamic nuclei in both PD and LBD. One histopathological study in 12 patients with PD showed that alfa‐synuclein deposition in the thalamus followed a specific pattern in which the intralaminar nuclei, the midline nuclei, and the limitans‐suprageniculate complex are most prominently affected, whereas the pulvinar is not prominently affected.^92^ A neuropathological study in patients with LBD who had visual hallucinations showed prominent neurodegeneration in the lateral pulvinar compared to patients with AD and healthy controls.^93^ Predominant alfa‐synuclein pathology in the intralaminar and midline nuclei may explain the typical cognitive profile in patients with LBD with attentional deficits and fluctuating alertness, given the role in these nuclei in arousal and attention (sections 4.4 and 4.5), as well as affective and behavioral symptoms. Neurodegeneration of the pulvinar in patients with LBD may explain the typically prominent visuoperceptual impairment (section 4.3.2) and predisposition to visual hallucinations through disinhibition of the primary visual and visual association cortices. Even though these studies do not provide evidence for a causal relation between alfa‐synuclein pathology in the thalamus and cognitive and behavioral symptoms, the convergence between the location of synuclein deposition in specific thalamic nuclei and their corresponding function does suggest a role of thalamus pathology in explaining cognitive and behavioral symptoms in PD and LBD.

#### 6.1.3 Vascular pathology

There is a large body of literature implicating the thalamus as a critical structure in vascular dementia. Case series from the mid and late 20th century already suggested that the thalamus was a strategic infarct location,^10^, ^36^ that is, a region where single small infarcts could cause major cognitive decline, and this has been corroborated by recent large‐scale lesion‐symptom mapping studies.^10^, ^94^ Recent lesion‐symptom mapping studies have not only confirmed the role of thalamic lesions in vascular dementia but also provided higher anatomical precision and demonstrated that in particular infarcts in the left anterior and dorsomedial nuclei of the thalamus carry a high risk of post‐stroke cognitive impairment,^94^ which can be explained by the prominent role of these nuclei in many cognitive functions (sections 4.1 and 4.2). White matter hyperintensities (WMHs) and lacunes in the anterior thalamic radiation, which connects the anterior and dorsomedial thalamic nuclei with frontal cortical regions, have also been associated with impairment in multiple cognitive domains.^22^, ^95^, ^96^ Apart from depriving anatomically connected remote brain regions from thalamic input, small thalamic infarcts can also disrupt widespread functional brain networks (eg, ipsilateral reduced activation of the DMN^97^) and ipsilateral cortical metabolic changes (eg, ipsilateral cortical hypoperfusion^98^) as possible mechanisms for the profound cognitive impact of thalamic infarcts.

### 6.2 Clinical implications and future perspectives

**FIGURE 3:**
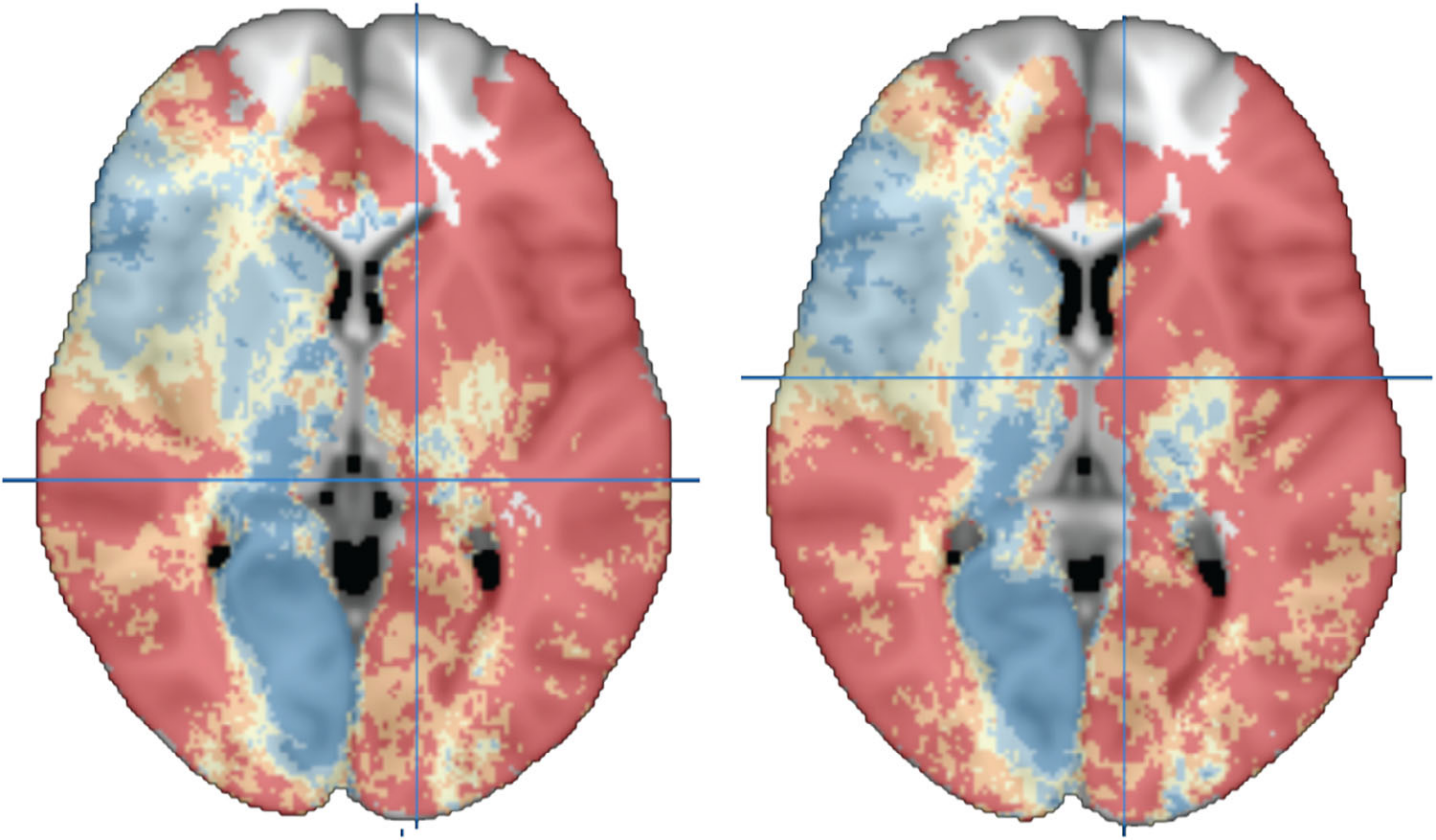
The previously published visual scale for rating the location impact score, which consists of five risk strata for post‐stroke cognitive impairment (PSCI) based on infarct location.^94^ These two transversal slides were extracted from the three‐dimensional (3D) map published online at https://metavcimap.org/features/software‐tools/location‐impact‐score/ to highlight the thalamus. Dark blue regions correspond to lowest risk and red regions to highest risk of PSCI. Looking specifically at the thalamus, several infarct locations are predictive of a high risk of PSCI, prominently involving the anterior and dorsomedial nuclei and pulvinar, and PSCI risk is higher in most left thalamic regions compared to the corresponding right thalamic regions. In the left panel, the crosshairs are located at the left pulvinar, MNI coordinates (*x*, *y*, *z*) −13, −31, −3. In the right panel, the crosshairs are located at the left anterior thalamic nuclei, MNI coordinates (*x*, *y*, *z*) −8, −6, 6. The 3D map can be accessed online for the evaluation of scans of individual patients against the impact score.

At present, the clinical implications of considering thalamic lesions or pathology in cognitive decline and dementia are most straightforward in vascular cognitive impairment. The presence of thalamic infarcts can be readily assessed with MRI and, even though individual thalamic nuclei usually cannot be distinguished reliably with routine imaging protocols, infarct location in relation to major nuclear divisions (eg, anterior, dorsomedial parts of the thalamus or the pulvinar) and risk of cognitive impairment can be ascertained (Figure 3).^94^ In addition, considering the location of thalamic infarcts in relation to thalamic functional anatomy (as discussed in section 4) may help determine whether a visible thalamic lesion is a plausible explanation for a patient's cognitive and behavioral symptoms.

Despite evidence for an important role of thalamus pathology in explaining cognitive decline and behavioral symptoms in patients with neurodegenerative dementias, this currently does not appear to have direct implications for diagnosis and treatment in clinical practice. This may in part be due to the fact that the role of the thalamus in AD and other neurodegenerative etiologies has received relatively little attention. The early deposition of AD pathology in limbic thalamic nuclei and its relation to cognitive and behavioral symptoms (evidence summarized in section 6.1.1) may have implications for network models of the functional impact of AD pathology and for disease propagation models that incorporate a potential role of prion‐like spreading of misfolded amyloid beta and tau proteins in the brain connectome.^99^, ^100^ Emerging therapeutic implications that are being explored include deep brain stimulation of thalamic nuclei in order to ameliorate cognitive decline and behavioral symptoms in patients with AD, given its location both upstream and downstream from the hippocampus and retrosplenial cortex (structures implicated in AD‐related memory decline)^85^ and its role as an integrative hub in many distributed brain networks including the DMN.^101^ At present, evidence that thalamic deep brain stimulation (targeting intralaminar nuclei, midline nuclei, or anterior thalamic nuclei) indeed has sufficient beneficial effects on cognitive symptoms in patients with AD to justify such invasive treatment is insufficient and further research is needed.^102^ Furthermore, it has been suggested that volumetry of specific thalamic nuclei might have diagnostic value, based on the observation that dementia etiologies differentially affect specific thalamic nuclei.^103^ However, in our view, it seems unlikely that advanced structural injury markers such as thalamus atrophy will play a large role in diagnosing dementia etiology, as this increasingly relies on more specific biomarkers of the underlying disease.^5^ Volumetry of thalamic nuclei may therefore mainly be of interest for advanced clinical research on pathophysiology, progression patterns of neurodegeneration, and perhaps interventions in the setting of dementia, in particular for etiologies for which no reliable in vivo diagnostic biomarkers are currently available.

## 7 SUMMARY

The thalamus forms a central part of numerous widespread brain networks and performs a critical role in relaying, modulating, and integrating sensory and (sub)cortical inputs. Accordingly, the thalamus is involved in nearly all aspects of cognitive functioning and behavior (sections 3, 4, and 5). In the context of dementia, the relevance of thalamus pathology in vascular cognitive impairment has been firmly established, and these insights are now being incorporated into diagnostic and prognostic models that are likely to be further improved in the future (sections 6.1.3 and 6.2). By comparison to vascular cognitive impairment, the thalamus has received relatively little attention in AD research (sections 6.1.1. and 6.2). The available evidence suggests that AD pathology that accumulates in the thalamus (in particular involving the anterior thalamic nuclei, midline nuclei, and intralaminar nuclei) might cause widespread brain network dysfunction, which contributes to cognitive and behavioral symptoms in AD. Further research is needed to elucidate the interplay between local AD pathology in the thalamus, hippocampal formation, and neocortex in causing brain network dysfunction and to determine the relative contributions of thalamus pathology (independent of, for example, hippocampal and cortical pathology) to cognitive and behavioral symptoms. In particular, longitudinal imaging studies in which amyloid/tau deposition, structural brain changes, brain connectomics, and cognitive functioning are repeatedly assessed in patients with AD pathology and healthy controls may help to address these questions. Such studies will primarily have implications for improving our understanding of the role of AD pathology in the thalamus and connected brain regions throughout the AD disease process. Whether these insights may ultimately have therapeutic consequences remains to be determined. One particular therapeutic avenue that might be further explored is whether neuromodulation of thalamic nuclei could ameliorate cognitive and behavioral symptoms in patients with AD.

## CONFLICT OF INTEREST STATEMENT

The authors have no conflicts of interest to disclose.

## Supporting information


Supporting Information
